# A protocol for laser microdissection (LMD) followed by transcriptome analysis of plant reproductive tissue in phylogenetically distant angiosperms

**DOI:** 10.1186/s13007-019-0536-3

**Published:** 2019-12-16

**Authors:** Kimmo Kivivirta, Denise Herbert, Matthias Lange, Knut Beuerlein, Janine Altmüller, Annette Becker

**Affiliations:** 1 Institute of Botany, Justus-Liebig-University Gießen, Heinrich-Buff-Ring 38, 35392 Gießen, Germany; 2 Present Address: Freelance Trial Monitor and Manager for Non-Interventional Studies, Grolmanstr. 22, 10623 Berlin, Germany; 3 Rudolph-Buchheim-Institute of Pharmacology, Justus-Liebig-University Gießen, Schubertstraße 81, 35392 Gießen, Germany; 4 Cologne Center for Genomics (CCG), University of Cologne, Weyertal 115b, 50931 Köln, Germany

**Keywords:** Laser microdissection (LMD), RNA-seq, Evo-devo, Development, Cryosectioning, Non-model species

## Abstract

**Background:**

Plant development is controlled by the action of many, often connected gene regulatory networks. Differential gene expression controlled by internal and external cues is a major driver of growth and time specific differentiation in plants. Transcriptome analysis is the state-of-the-art method to detect spatio-temporal changes in gene expression during development. Monitoring changes in gene expression at early stages or in small plant organs and tissues requires an accurate technique of tissue isolation, which subsequently results in RNA of sufficient quality and quantity. Laser-microdissection enables such accurate dissection and collection of desired tissue from sectioned material at a microscopic level for RNA extraction and subsequent downstream analyses, such as transcriptome, proteome, genome or miRNA.

**Results:**

A protocol for laser-microdissection, RNA extraction and RNA-seq was optimized and verified for three distant angiosperm species: *Arabidopsis thaliana* (*Brassicaceae*), *Oryza sativa* (Poaceae) and *Eschscholzia californica* (Papaveraceae). Previously published protocols were improved in processing speed by reducing the vacuum intensity and incubation time during tissue fixation and incubation time and cryoprotection and by applying adhesive tape. The sample preparation and sectioning of complex and heterogenous flowers produced adequate histological quality and subsequent RNA extraction from micro-dissected gynoecia reliably generated samples of sufficient quality and quantity on all species for RNA-seq. Expression analysis of growth stage specific *A. thaliana* and *O. sativa* transcriptomes showed distinct patterns of expression of chromatin remodelers on different time points of gynoecium morphogenesis from the initiation of development to post-meiotic stages.

**Conclusion:**

Here we describe a protocol for plant tissue preparation, cryoprotection, cryo-sectioning, laser microdissection and RNA sample preparation for Illumina sequencing of complex plant organs from three phyletically distant plant species. We are confident that this approach is widely applicable to other plant species to enable transcriptome analysis with high spatial resolution in non-model plant species. The protocol is rapid, produces high quality sections of complex organs and results in RNA of adequate quality well suited for RNA-seq approaches. We provide detailed description of each stage of sample preparation with the quality and quantity measurements as well as an analysis of generated transcriptomes.

## Background

Understanding the function of genes requires knowledge about the localization and timing of their expression. Consequently, the analysis of expression pattern of genes during plant development, in different mutant backgrounds, after exposure to stressors, in changing growth conditions etc. has been utilized to unravel novel gene functions and to attribute genes to genetic networks.

Several methods were developed over the years to provide quantitative or semi-quantitative information on gene expression, such as Northern Blotting, where total RNA is fixed to a membrane and the target gene sequence is labelled and hybridized to the immobilized RNA. More indirect methods to detect RNA abundance include RT-PCR and qRT-PCR as RNA quantification is calculated from increase of PCR amplification products. The method offering the highest spatial and temporal resolution of gene expression analysis is RNA in situ hybridization for which a, e.g. digoxigenin-labelled antisense probe is hybridized to either whole-mount tissue or tissue sections but it is unsuited for transcript quantification [1].

These methods all have in common that single or few genes at the time can be analysed without the need for genetic manipulations. In contrast, reporter gene studies depend on genetic manipulation of the target species as the promoter:reporter gene combination requires transfer into the plant. This method also allows a high spatial and temporal resolution and, in addition, live-cell imaging and has become invaluable to follow gene expression over time. However, many species are recalcitrant to genetic transformation and thus this method is not applicable for a large fraction of plants [2].

Microarray experiments allow the simultaneous quantification of thousands of genes’ expression but require at least partially sequenced genomes or transcriptomes from which the costly gene chips are generated. In the past years, RNAseq has become the most popular method to analyse gene expression changes across the entire transcriptomes in a large variety of species. However, this method also requires tissue collection for RNA extraction, with high quality requirements for the RNA to be sequenced, whereas high spatial and temporal resolution is as challenging as for the other methods [3].

Here we describe a method of cryo-sectioning and laser microdissection (LMD) of plant tissue followed by RNAseq (LMD-RNAseq) that is effective in phylogenetically distant plant species allowing the parallel analysis of whole transcriptomes while offering high spatial resolution. Several reliable protocols have been published previously, e.g. for *A. thaliana*, *Citrus clementina* (clementine) and *Solanum lycopersicum* (tomato) [4–8]. However, these all differ significantly from each other and their adaptation to different tissues or organisms is time consuming. For example, sample preparation described in several protocols involves a lengthy period of dehydration and fixation with several steps taking up to a week and leading to alteration of the RNA environment within the cells and tissues. Alternatively, comparably faster, more general methods for plant tissue are available [9, 10] but they might prove insufficient for small heterogeneous samples including several different tissue types. The method described here reliably produces high-quality RNA from laser microdissected floral tissue from the phylogenetically distant species *A. thaliana* (Brassicaceae, core eudicot), *E. californica* (Papaveraceae, early branching eudicot) and *O. sativa* (Poaceae, monocot).

We optimized and accelerated a method based on fixation and cryo-sectioning of plant tissue and combined this with the use of LMD film [11] to produce accurate sections of flowers of different species and developmental stages. Anatomical retention of the sample sections is improved by adding LMD film which facilitates subsequent laser microdissection of target tissues. Alteration to the RNA environment of the samples as well as the impact to the RNA quality and quantity are reduced as the different steps of fixation and pre-treatment have a lesser effect on the target tissue compared to the lengthy process of dehydration and paraffin embedding generally used for high detail sectioning. This protocol as well as the details of reporting the quality of intermediate steps will provide other researchers requiring transcriptome analysis with high spatial resolution with a workflow and reporting guidelines for LMD-RNAseq in model and non-model plant species in the clade of *Angiospermae*.

## Results

**Fig. 1:**
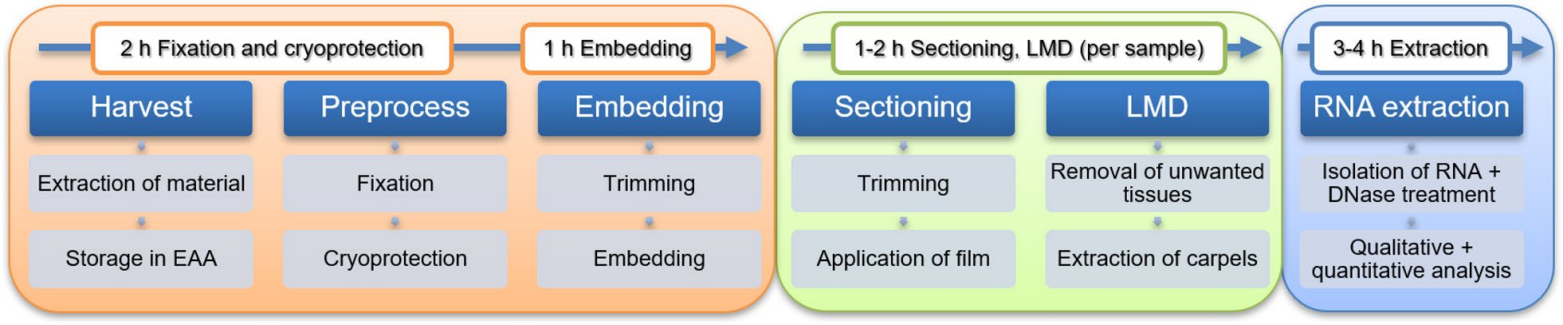
Schematic representation of the complete LMD workflow illustrating the order of steps and time consumption of the method from harvesting the tissue to analysing the RNA. Samples can be frozen and stored for further processing at the end of each of the three phases of sample preparation

A method based on cryosectioning and laser-microdissection followed by RNA extraction and RNAseq was optimized to obtain information on transcriptionally active genes from the gynoecia of diverse angiosperms species. We provide a protocol for this method (Fig. 1) that allows transfer of the method to a wide array of phylogenetically distant angiosperm species.

**Fig. 2:**
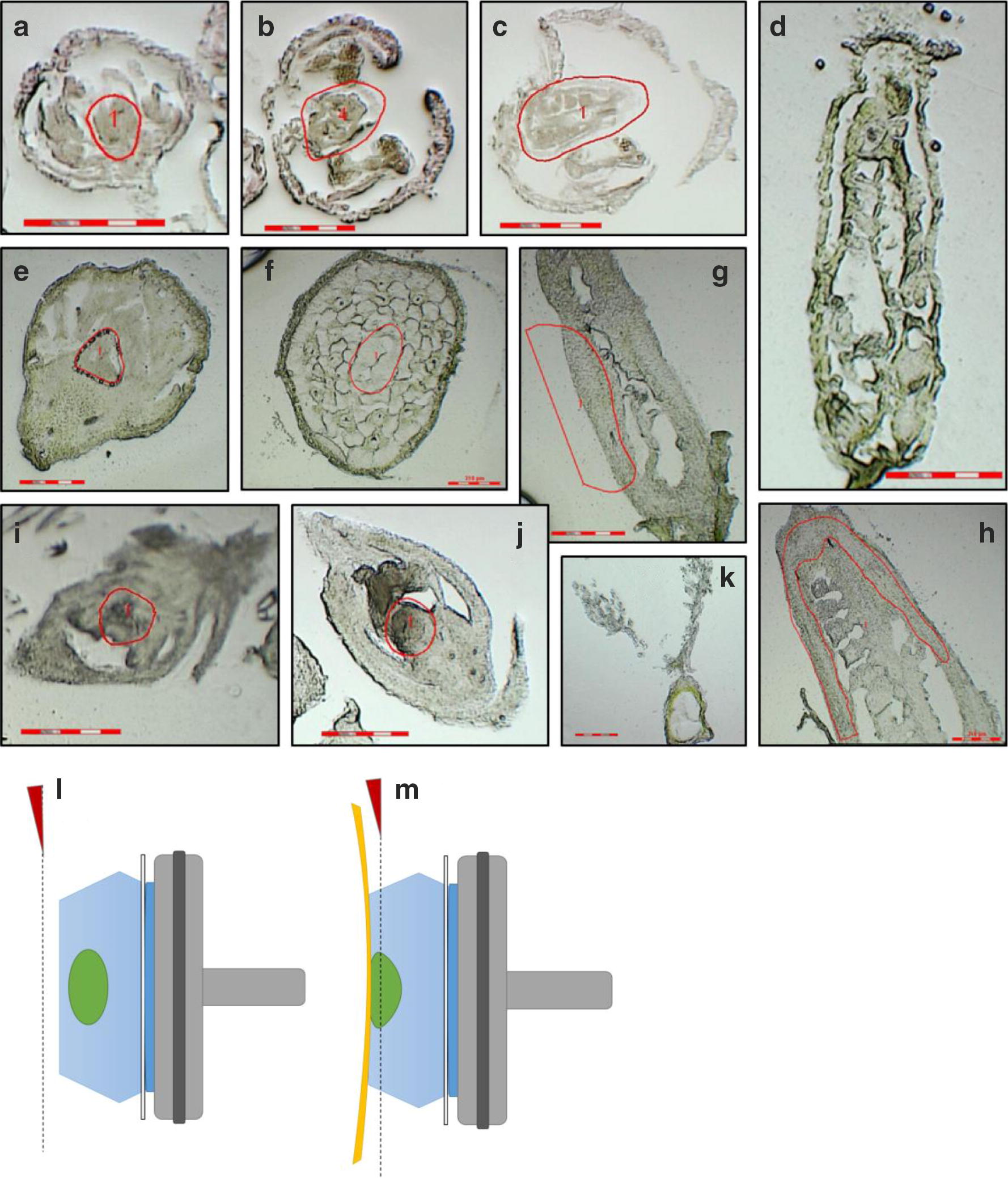
Representative tissues selected from three different species (*E. californica*, *A. thaliana*, *O. sativa*) which were cryo-sectioned and observed under an LMD microscope. Predetermined LMD elements to be excised and collected are shown in red in all sections except for **d** and **k**. For sections **d** and **k**, the whole section was excised and collected in pieces after removal of the ovules. **a**–**d** *A. thaliana* buds, **e**–**h** *E. californica* buds; **i**–**k** *O. sativa*. Tissue was collected at four different (three for *O. sativa*) developmental stages (Table 1): Initiation of carpel development (**a**, **e**, **i**); elongation of carpel walls (**b**, **f**, **j**); during meiosis (**c**, **g**) and after meiosis (**d**, **h**, **k**). **a** Stage 6; **b** stage 9; **c** stage 11; **d** stage 13, stages according to Smyth et al. [13]. **e** Stage 5; **f** stage 7; **g** stage 8, **h** stage 9, staging according to Becker et al. [12]. **i** stage 6; **j** stage 7; **k** stage 9, staging according to Ikeda et al. [15]. Sections are all transversal except in **f** (vertical), scale bar is 300 µm. **l**, **m** Illustrations of frozen O.C.T. compound blocks adhered to sample base before (**l**) and after (**m**) trimming the excess compound and adhering the adhesive film on the sample block. Grey, sample holder; green, embedded sample; blue O.C.T. surrounding the sample; yellow: LMD film; dotted line, sectioning plane

**Fig. 3:**
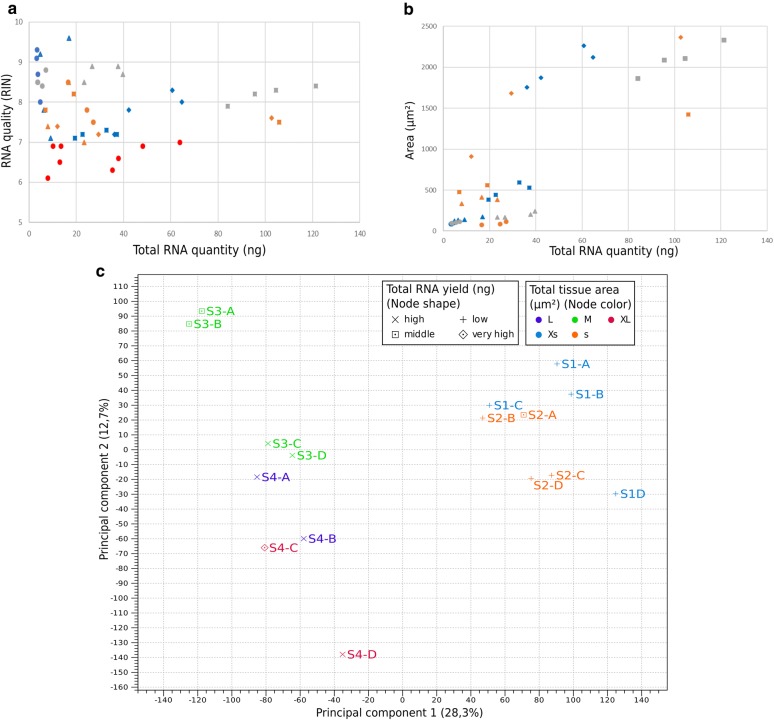
Correlations of RNA quality and quantity, tissue size, sample replicates of RNA of different species (orange: *E. californica,* blue*: A. thaliana,* grey*: O. sativa,* red: excluded samples) and developmental stages (symbols in figures A and B, stage 1: circle; stage 2: triangle; stage 3: square; stage 4: diamond). **a** RNA quality (RIN) in relation to yield [ng]. **b** relation of area of tissue used for extraction [mm^2^] to the RNA quantity [ng]. **c** Principal component analyses of the *A. thaliana* RNA Seq samples. S1—S4 show the different developmental stages, **a**–**d** corresponds to the four replicas per stage. Node shapes show total RNA (ng): low = 4–15 ng; middle = 16–30 ng; high = 31–45 ng; very high ≥ 46 ng. Different colors correspond to the total area of the collected samples (mm^2^): XS = 50–100 µm^2^; S = 101—300 µm^2^; M = 301—1000 µm^2^; L = 1001—2000 µm^2^; XL = 2001—3000 µm^2^

**Fig. 4:**
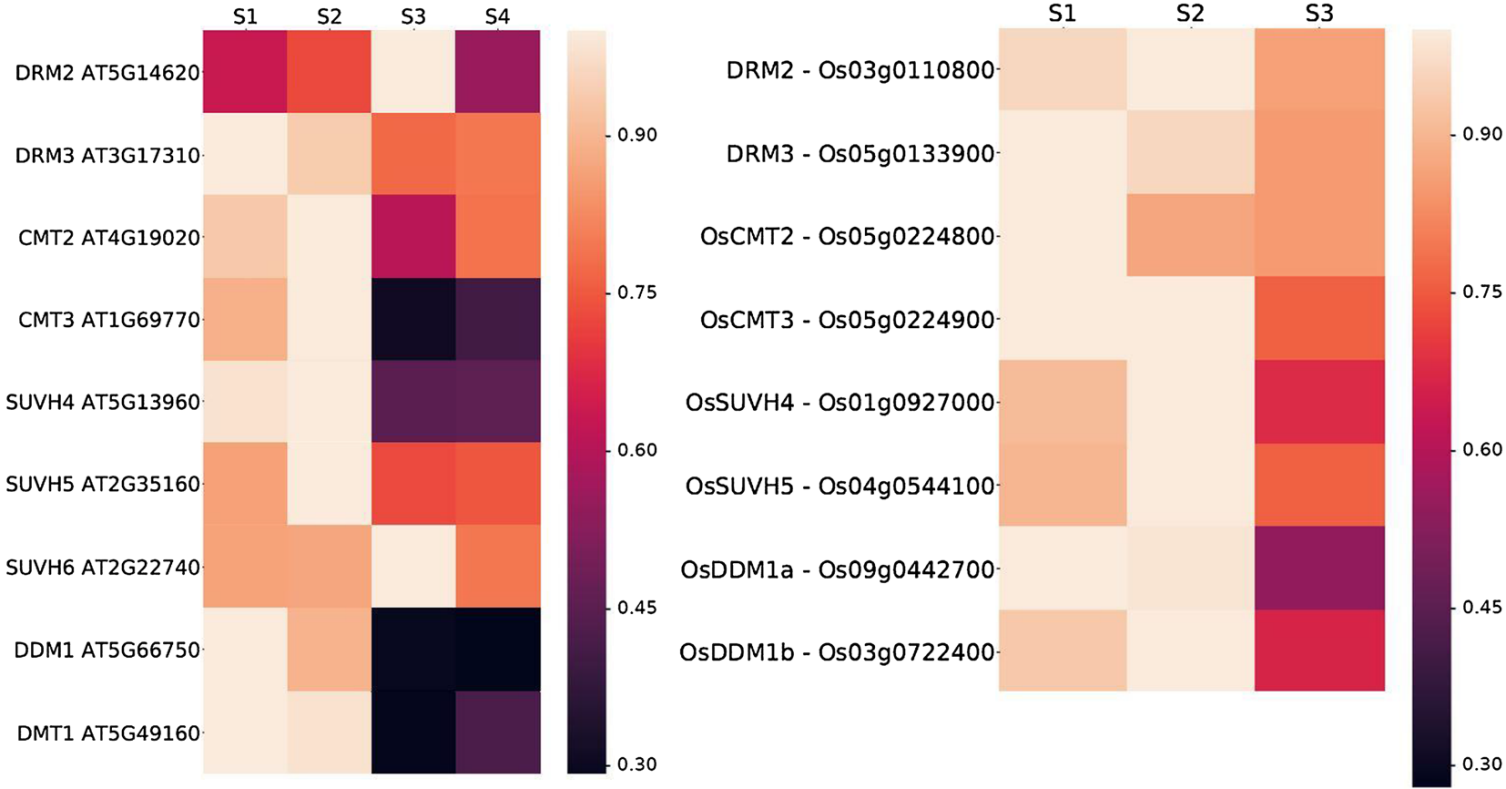
Chromatin-related genes as an example for differential gene expression. Heat map of the expression patterns of several DNA methylation related factor genes across different stages of carpel development (S1-S4) in *A. thaliana* and *Oryza sativa* (S1-S3). Gene names are shown on the left side, accompanied by TAIR and *O. sativa* IDs. Stages of carpel development are shown on top. Light yellow colors indicate a high/strong expression, whereas darker red to purple colors indicate low expression, the highest TPM value of a gene was set to 100% (1.00)

Three species from distant angiosperm families were chosen to validate the protocol. Sections were prepared from buds of *A. thaliana* (Col-0), *E. californica* and *O. sativa* (ssp. Dongjin) at four (three for *O. sativa*) different developmental stages (Table 1): (1) initiation of carpel development (Fig. 2a, e, i); (2) elongation of carpel walls (Fig. 2b, f, j); (3) during female meiosis (Fig. 2c, g) and (4) after female meiosis (Fig. 2d, h, k). Corresponding stages for each species were defined after Becker et al. [12] for *E. californica*, Smyth et al. [13] and Armstrong and Jones [14] for *A. thaliana,* and Ikeda et al. [15] for *O. sativa* and correlated to the four carpel stages analysed here (Table 1). Flower buds were fixed, cryoprotected, embedded and sectioned according to the protocol described. 10 µm sections from each species and developmental stage were documented under a Leica DM6000 B microscope (Fig. 2). Bud lengths ranged from 0.3 mm (Fig. 2a: initiation of carpel development in *A. thaliana*) to over 10 mm (Fig. 2h: post-meiotic growth stage in *E. californica*).

**Table 1:** Correlation of the four (three for *O. sativa*) developmental stages of species used in this study with floral developmental stages described elsewhere

	Stage 1: carpel initiation	Stage 2: elongation of carpel walls	Stage 3: during meiosis	Stage 4: after meiosis	Source
*E. californica*	0.39–0.65 mm BD: stage 5, carpel initiation	1.65–2.25 mm BD: stage 6, microsporangia initiate	2.3–2.8 mm BD: stage 8: male meiosis	3.5–5.5 mm BD: stage 9, female meiosis	Becker [12]
*A. thaliana*	0.3 mm BL: stage 5, sepals enclose bud	0.4 mm BL: stage 9, petal primordia stalked at base	0.5 mm BL: stage 11, stigmatic papillae appear	0.7–1 mm BL: stage 12, petals level with long stamens	Smyth et al. [13]; Armstrong and Jones [14]
*O. sativa*	0.9–1.5 mm IL: stage 6, differentiation of glumes	1.5–40 mm IL: stage 7, differentiation of floral organs	–	40–220 mm IL: stage 8. rapid elongation of rachis and branches	Ikeda et al. [15]

*BD* bud diameter, *BL* bud length, *IL* inflorescence length

*Arabidopsis thaliana* stage 3 and 4 stages were not fixed or cryoprotected prior to sectioning and for all species, stage 3 and 4 gynoecia were collected without ovules. At least three biological replicates were prepared for each stage of the development. LMD film (Fig. 2l, m) was used to improve the structural integrity of the sections, especially for the early stages and when sectioning through more robust organs leads to folding and/or dislocation of tissue.

### Factors determining RNA quality and yield

Determining RNA quality and quantity following the extraction is crucial for a qualified selection of samples for subsequent sequencing and was assessed by RNA integrity number (RIN) measurements. Some examples for RIN measurements are shown in Additional file 1: Fig. S1 and all measurements are summarized in Additional file 1: Tables S1–S3. Samples with RIN less than 7.0 were excluded from the analysis (Additional file 1: Table S4). Interestingly, our comparative analysis showed that RNA quality is mainly dependent on the species and only to a lesser extent on the developmental stage (Fig. 3 and Additional file 1: Tables S1–S3): *O. sativa* samples showed only little variation and had a RIN between 7.9 and 8.9. *E. californica* samples had a low quality when compared to *A. thaliana* or *O. sativa* (max. RIN of 8.5 with several samples discarded because of a RIN below the threshold, Additional file 1: Table S4) with samples of stage 1 and 2 show a higher RIN than later stages. *A. thaliana* samples show the largest distribution (RIN 7.1 to 9.6) with the highest RIN numbers achieved for stage one and two (Fig. 3a, Additional file 1: Tables S1–S3). We found no direct relationship between RNA yield and quality (Fig. 3a).

RNA yield is dependent on the amount of tissue used for the extraction and the developmental stage (Fig. 3b and Additional file 1: Tables S1–S3). Unsurprisingly, younger stages generally yield more RNA per dissected tissue area than older stages because of higher cell density, and larger dissected tissue areas yield more RNA. However, yield is also species dependent, for example, the *E. californica* stage 1 samples yield up to six times more RNA per dissected tissue area than the same *A. thaliana* stage samples (Fig. 3b). Moreover, we found that especially in *E. californica* large variation in the yield/dissected tissue area ratio (Additional file 1: Table S3), the most dramatic comparison being a stage one sample (S1-19) that yielded 16.6 ng RNA from 77 µm^2^ dissected tissue area while a stage two sample (S2-13) yielded only 7.9 ng from 335 µm^2^ dissected tissue area.

These results suggest that sample preparation and RNA extraction from microdissected tissue is dependent on several factors: RNA quality depends to a large extent on the species whereas quantity is dependent on the dissected tissue area, stage, and species.

### Sample variance analysis reveals distinct groups

Our original hypothesis was that high RNA quality positively correlates with high read numbers and to test this, we performed a PCA with the *A. thaliana* transcriptomes to visualize the variance in the samples based on Additional file 1: Table S1 and to identify reasons for sequencing success (Fig. 3c). PCA1 explains 28.3% of the variance and PCA 2 explains 12.7% respectively (Fig. 3c). The observed pattern shows two main groups. One group including the samples of the first two developmental stages (for stage description, see Table 1, samples S1A-C; stage 2, sample S2A-D), and the other group includes the samples of the stage 3 (samples S3A-D) and stage 4 (samples S4A-D).

Each main group can be further subdivided into smaller groups. The pattern of those subgroups consists, for the early developmental stages of samples of two subgroups: 169-A1, 167-A1, 170-A1, 180-A2 and 181-A2, for subgroup 1 and includes the samples 182-A2, 160-A2 and 161-A1 for subgroup 2. The group for the later developmental stage can be divided into four subgroups. Subgroup 1: 192-A3 and 193-A3; subgroup 2: 194-A3, 191-A3 and 188-A4; subgroup 3: 189-A4 and 190-A4; and Subgroup 4: 187-A4. The subgroups within the main groups show no distinct pattern. Several metadata were included to reveal the observed pattern (Additional file 1: Table S1), but none of the included metadata could completely explain the subdivision into smaller groups. Hence total RNA (ng), quality of RNA (RIN) and size of the samples (area) explained some more structure. PCA data for *E. californica* do not define obvious groups and those for *O. sativa* show clearly distinct groups based on the stages (Additional file 1: Fig. S2). Interestingly, a stage specific grouping could be observed only in one species out of three.

### Sequencing and data validation

We aimed to sequence 30 Mio. paired-end reads per library but the sequenced raw reads were between 30 Mio. and 59 Mio. reads (Additional file 1: Tables S1–S3). The respective reads were then mapped onto *A. thaliana* and *O. sativa* genomes to identify the number of mapped and unmapped reads. Of the mapped reads, rRNA, protein coding and intergenic reads were identified. The quality of the *E. californica* genome [16] proved insufficient for high-confidence read mapping and thus further characterization of the transcriptomes was omitted. rRNA reads were between 62 and 86% of all reads suggesting that rRNA depletion is not uniformly successful. The percentage of protein coding genes ranged between 18 and 37% in the libraries. We then scored the transcripts with a TPM > 5 and identified between 14,425 and 15,687 transcripts expressed in each library (Additional file 1: Tables S1–S3). The raw reads were deposited in Genbank with the Bioproject accession numbers PRJNA549137. Sample distance correlation was calculated for at least three replicates per species/developmental stage (Additional file 1: Figure S3).

The transcriptomic data was corroborated by comparing results from RT-PCR experiments of *A. thaliana* to the expression values in the transcriptomes. Because of the overall low RNA yield of the LMD experiments which is inadequate for RT-PCR, RNA was extracted from complete early (Stage 1) and late (stage 4) buds. Gene expression analysis of the qRT-PCR with the TPM values of the transcriptomes was carried out to enable some degree of comparison with our stage one and stage four *A. thaliana* transcriptome data. Further, we compared our data with corresponding samples (young bud at stage 2: “elongation of carpel walls” and carpel of stage 4: “after meiosis”) in the Klepikova atlas [17]**.** We selected two genes related to the carpel development: *CRC *[18], and *HAT1 (*or *JAIBA* [19] and the two chromatin remodellers *DDM1* [20] and *CMT3* [21]. The expression between the three sources roughly followed similar patterns (Additional file 1: Figs. S5, S6 and Tables S5, S6 and S7), with *CRC* strongly expressed in young but hardly expressed in the older samples, and *DDM1* and *DDM3* showing stronger expression in younger than in older samples. *HAT1/JAIBA* shows a stronger expression in older than in younger stages in the transcriptome comparisons and almost similar expression in both stages in the qRT-PCR. Differences were most noticeable in the strength of expression most likely due to the fact that the samples from different sources were prepared in different ways. Taken together, our transcriptome analysis is in line with qRT-PCR results and previously published data relying on fewer tissues and stages.

### LMD-RNAseq of angiosperm carpels provides a valuable resource for the discovery of development stage-specific genes

We were interested in quantifying the sensitivity of our method and identified genes that are expressed in one or several specific stages of the three species we worked with (Additional file 1: Table S7). Thus, we defined patterns based on presence (TPM > 5) or absence (TPM < 5) of a gene’s expression in the carpel development transcriptomes. In *A. thaliana* transcriptomes, 143 genes were specific to stage 1, 225 genes specific to stage 2, 427 genes specific to stage 3, and only 74 genes specific to stage 4. In *O. sativa*, we identified 518 genes specific to stage 1, 328 genes specific to stage 2, and 789 genes specific to stage 3. In *E. californica*, we identified 507 genes specific to stage 1, 688 genes specific to stage 2, 895 genes specific to stage 3, and 837 genes specific to stage 4. Further, we identified genes specific to all possible patterns (e.g. “early” [stage 1 and 2 specific genes] versus “late” [stage 3 and 4 specific genes]) in all species The pattern poorest in gene number is the stage 1 and stage 4 specific genes in *A. thaliana* with only six members and the pattern richest in gene number is stage 1 and stage 2 specific genes of *O. sativa*, ecompassing 1147 members.

We were then interested to know if the gynoecium stages we collected resulted in expression differences of a functionally related group of genes. As DNA methylation is thought to regulate cellular differentiation also in plant reproduction [20-25], we chose to analyse the expression of nine genes present in the gynoecium transcriptomes coding for eight DNA methyltransferases and *DDM1*, encoding a DNA methylation regulating SWI2/SNF2 family chromatin remodeler (Fig. 4) [26, 27]. Each of these genes shows a distinct expression pattern: *DRM2* and SUVH6 show their strongest expression in stage three, but most other genes are expressed at a higher level in stages one and two and at lower level at stages three and four. All genes show an expression pattern distinct in all four stages (Fig. 4a) suggesting that the *A. thaliana* gynoecium transcriptomes provide a valuable resource to identify stage-specific gene expression. To learn if the gene expression pattern of the *A. thaliana* chromatin remodelers is conserved in their rice orthologs we identified the putative *A. thaliana* orthologs in *O. sativa* (Fig. 4b). While we identified single rice orthologs for *DRM2*, *DRM3*, *CMT2*, *CMT3*, *SUVH4*, and *SUVH5*, no orthologs expressed in the carpel transcriptomes were found for *SUVH6* and *DMT1*, while two rice orthologs were identified for *DDM1*. All analysed rice genes showed their strongest expression in the first two stages of carpel development and a lower expression in the latest stages and their expression is comparable to their *A. thaliana* orthologs. However, *DRM2* is the exception as its expression in *A. thaliana* is strongest in stage three while its rice ortholog shows its strongest expression in the first two stages. Taken together, we can show differential, stage-specific expression of chromatin remodels of *A. thaliana* and *O. sativa* in a pattern largely conserved between orthologs of the two species.

## Discussion

Here we describe a widely applicable method for laser microdissection of tissue for RNA extraction and subsequent RNAseq with stage specific tissue collection of gynoecia. This method combines rapid fixation with cryo-sectioning and application of LMD film and is a fast alternative to the process of dehydration and paraffin embedding or other lengthy methods of cryo-sectioning as the fixation and cryoprotection for this method can be carried out within a single day. By reducing the vacuum intensity and incubation time spent on fixation and cyoprotection, we could reduce the time spent on the complete protocol to just two days. The improvement in speed means the degrading elements have less time to affect the RNA. RIN quality of over 9.0 was measured from some of the samples and the large majority of the samples exceeded RIN 7.0. Adhering the whole organ to the LMD film prior to sectioning remedies a variety of challenges posed by the complexity of the samples: the smallest of the early stage buds are only 0.3 mm in size hindering the location of the sample, even minor breakdown or loss of histological retention of the bud during cryo-sectioning would make it difficult to clearly differentiate gynoecium tissue from the surrounding organs. In later stages, maturing organs of different firmness, air pockets, porous structures and hairs would occasionally lead to breaking, dislocation or folding of structures such that these sections could not be used further. Moreover, the addition of the LMD film simplified the process of cryo-sectioning: LMD membrane slides could be completely replaced by the adhesive LMD film. For large buds, fixation and cryoprotection can in some cases be omitted if the LMD film is used to fix the samples. Fixation was omitted for the later stages of *A. thaliana* samples leading to a lower histological quality. Removal or reduction of intensity of vacuum treatments such as fixation and cryoprotection or dehydration prior to paraffin embedding may prevent the dislocation of cellular fluids and components into the fixative solution therefore giving more accurate representation of the RNA expression profile of the target tissues [11]. Section thickness was tested from 5 to 15 μm and resulted in similar histology and structural integrity. The protocol was tested on gynoecium developmental stages ranging from inception to maturity. Because the gynoecium is surrounded by other floral organs, penetration of the fixative is difficult to achieve. However, as most other plant tissues are less protected by other organs (with the exception of the shoot apical meristem), our protocol will also work for other, less complex organs. For smaller and/or and more complex samples, LMD film improves the structural integrity as shown for highly complex whole rat embryos [11].

Integrity of the RNA samples varied between 6.1 and 9.6. Using the protocol established in this work. On average these numbers are comparable to the integrity resulted by some of the recent methodology with the highest integrity in *A. thaliana* (RIN 8.0 to 8.10 [28]) and *O. sativa* (RIN 8.5 to 8.5 [29]). Only *E. californica* samples averaged for a notably lower RIN of 7.3. We decided to set the integrity threshold to RIN 7.0 to ensure the high quality of RNA-seq output, although RIN as low as 5.7 has been reported to produce high quality reads [7]. Interestingly, we observed that RNA integrity did not directly correlate with read count or other qualities of RNA-seq data (Additional file 1: Tables S1–S3). Yield and concentration of RNA samples varied between species, with *E. californica* providing the highest RNA yield per unit area. RNA concentrations of *E. californica* samples from early stages were in the range of what has been published for tomato [9]. Also, previously published work shows that high RIN did not correlate to high yield, and unexpectedly, there was no strong evidence that younger tissues provide higher RNA quality [30]. Several factors may have had an effect on RNA quality: (i) pre-treatment was omitted from the two later stages of development in *A. thaliana* possibly leading to lesser quality. Other factors might also play a role such as (ii) presence of secondary metabolites interfering with RNA isolation or subsequent steps, (iii) varying abundances of RNases, (iv) differences in the kits used for RNA isolation and library preparation. However, previous work for standard, non-LMD treated samples has shown that maximizing RIN will increase transcriptome sequencing success but transcriptome sequencing is also robust to variation in RNA quality [30].

A high proportion of raw data was lost to rRNA reads (63% to 86%) which were later discarded during the assembly of the transcriptomes. Most likely, this was due to the choice of rRNA depletion method with the NuGEN Ovation® kit, which is was designed to target mammalian rRNA. However, this kit produced better results in rice previously (11–53%) [31]. Other kits for rRNA depletion are commercially available (i.e. RiboMinus™, Thermo Fisher Scientific, Waltham, Massachusetts, USA) but these often require a high concentration of RNA which is difficult to achieve with LMD or are unavailable for most plant species. Kits for targeted transcript depletion (NuGEN Ovation® SoLo RNA-Seq: AnyDeplete, San Carlos, California, USA) have only recently become available and could offer a solution for the unwanted reads in low quantity samples to further improve sequencing depth and thus improve the detection of rare transcripts like e.g. transcription factors.

## Conclusions

Here we provide a protocol for LMD followed by RNAseq that is applicable to complex, reproductive organs of flowering plants and and we show it to work successfully in the basal eudicot *E. californica*, the dicot *A. thaliana*, and the monocot *O. sativa*. The protocol is quicker than most protocols and encompasses fixation, cryoprotection, cryosectioning, laser microdissection, subsequent RNA extractions followed by library preparation, RNAseq and read assembly. While most previously published protocols are optimized for a single species only, we have developed a more universally applicable protocol that will be useful for researchers interested in fine scale temporal and spatial expression analysis based on RNA seq with diverse plant species.

## Materials and methods

A protocol based on cryosectioning and laser-microdissection was optimized and used for floral tissues from diverse species, followed by RNA extraction and RNA-seq (Fig. 1). The cryoprotective steps of the protocol were based on a modified protocol by Martin et al. [7]. The steps of cryo-sectioning were based on a modified protocol by Kawamoto and Kawamoto [11]. Generally, to ensure an optimal yield of intact RNA, work was carried out in aseptic conditions when possible and all working surfaces and tools were treated with RNase AWAY (Thermo Fisher Scientific, Waltham, Massachusetts, USA). For a more detailed protocol see Appendix: protocol (Additional file 2).

### Fixation and cryoprotection

For small, heterogenous samples with a multitude of different tissue types fixation and cryoprotection are recommended to achieve adequate histological quality for the following steps of the protocol. For some structurally homogenous samples these steps were omitted to speed up the process. Fresh flower buds were harvested and immediately submerged in a falcon tube filled with ice cold EAA (Ethanol (EtOH > 99%), Acetic acid, 3:1). Subsequent steps were carried out on ice. Vacuum was applied (300–400 mbar) for 10 min. EAA was discarded and the samples were transferred to a new falcon tube filled with 10% (w/v) sucrose solution prepared with PBS buffer (10× PBS buffer: 80 g NaCl, 14.4 g Na2HPO4·7H2O, 2 g KCl, 2 g KH2PO4, brought to 1 l with RNase free water, pH to 7.4). Vacuum was applied for 20 min (samples will first float in the solution but will slowly start sinking as the solution is penetrating the tissues). Falcon tube with the samples was placed on an undulating shaker in a refrigerator and the samples were let to incubate for at least one hour with a low speed. The solution was replaced with 20% sucrose solution prepared with PBS buffer and the vacuuming step was repeated for 20 min. The incubation step with the undulating shaker was repeated for one hour. Sunken buds were then prepared for embedding.

### Embedding

Excess material around the tissue of interest was trimmed prior to embedding. One to three samples similar in size were transferred to embedding moulds and completely submerged in O.C.T. compound (Sakura Finetek Europe B.V., The Netherlands). The buds were moved around the mould with forceps to ensure that the layer of sucrose solution was completely replaced with O.C.T. compound. The buds were moved to horizontal orientation in the bottom of the mould in which they were frozen at − 20 °C for 15–30 min. Once frozen, the samples were sectioned or placed in − 80 °C for long term storage.

### Sectioning

The cryochamber (Leica CM1850, Leica biosystems, Wetzlar, Germany) was adjusted to − 20 °C and the sample holder to − 25 °C. Required instruments were let to acclimate for 10 min in the cryochamber. Frozen sample block was attached to the sample base and to the pre-frozen sample holder. Excess O.C.T. compound and outer, unwanted layers were trimmed and discarded. Pre-frozen piece of LMD film type 2 (Section-lab Co. Ltd., Hiroshima, Japan) was adhered to the surface of the section and cut with a slow, constant speed. (Fig. 2m) 4–12 sections were cut per sample depending on the size of the samples and adhered to empty frame slides (Leica biosystems, Wetzlar, Germany).

### Laser-microdissection

Before dissection, the dissecting microscope [Leica DM6000 B microscope (Leica biosystems, Wetzlar, Germany) fitted with Crylas ftss 355–50 laser unit (CryLaS GmbH, Berlin, Germany)] was calibrated to optimize target fragment dissection and collection. The films were fully dried before starting the laser-assisted cutting, because moisture in the films may lead to warping of the membrane while cutting or fragments adhering to the film. Two sample collection tubes were attached to the sample collection tray: one for the samples and another for discarding unwanted material. Before starting the cut, the collection tube cap was filled with 20 μl of extraction buffer [PicoPure™ RNA Isolation Kit (Applied Biosystems™, Foster City, California, USA)]. The samples were located and the elements of the desired tissue were marked. Samples were cut with slow speed and the collection cap was inspected to make sure that the target tissues had fallen into the collection tube. The collection tube was detached from the tray, closed and vigorously vortexed for one minute. The tubes were then submerged in liquid nitrogen for further break down of protective cell structures.

### RNA extraction and quality/quantity assessment

RNA extraction for RNAseq was carried out using the PicoPure™ RNA Isolation Kit according to the manufacturer’s instructions and the samples were stored at − 80 °C. Multiple early stage samples were pooled together for a single extraction to increase the RNA concentration of the eluate. The quantity and the quality of the samples was measured using the Agilent bioanalyzer with Agilent RNA 6000 Pico Kit (Agilent Technologies, Santa Clara, California, USA).

### Library preparation and sequencing

RNA samples were pre-amplified using Nugen Ovation® RNA‑Seq System (PART NO. 7102) (NuGEN Technologies, San Carlos, California, United States) suitable for an input of 500 pg–100 ng of RNA and the cDNA generated was used for library preparation. Amplification and cDNA generation was conducted according to the manufacturer’s instructions. Library preparation was done with the Illumina Nextera XT kit (Illumina Inc., San Diego, California, USA) suitable for 1 ng DNA. The cDNA was measured before the preparation with Qubit and normalized to 0.2 ng/µl. 5 µl of dilution was used for the library preparation. Libraries were prepared according to the manufacturer’s instructions. Generated libraries were sequenced with Illumina HiSeq 4000. 9 samples were sequenced per lane generating approximately 33 million reads per sample. Paired-end sequencing was used for each sample with read length of approximately 76 bp.

### Assembly, heatmap, and principal component analysis (PCA)

Raw reads of paired end sequencing data were imported to CLC genomics workbench version 11.0.1. CLC Genomics workbench was used for quality test, trimming, RNA-seq and PCA analysis using the default parameters using information deposited in Additional file 1: Tables S1–S3. To evaluate gene expression patterns, genes related to chromatin remodeling in *A. thaliana* [27] and *O. sativa* were selected and their expression in TPM values within the transcriptomes were analyzed. *O. sativa* orthologs to *A. thaliana* chromatin remodelers were identified by using ProteinOrtho [32]. Their expression patterns were visualized in a heatmap created by an in-house python script [33] utilizing pandas [34], NumPy [35], Seaborn [36] and Matplotlib [37]. To evaluate how well the replicas of each probe set correlate with each other a sample distance matrix was calculated using the Bioconductor package Deseq2 in Rstudio [38].

### qRT-PCR

*Arabidopsis thaliana* Col-0 was grown in conditions described above. RNA was extracted with the NucleoSpin® RNA -kit (Macherey Nagel, Germany) from complete buds at growth stages 9 and 12 [13] and cDNA was generated using the RevertAid RT Reverse Transcription Kit (Thermo Fisher, Germany). Primer efficiency tests were carried out with an efficiency above 1.9 (Additional file 1: Table S5) selected for the analysis and three biological replicates were analyzed using the SYBR® Green based Luna Universal qPCR Mastermix (NEB, Germany) on a LightCycler® 480 Instrument (Roche, Germany).

## Supplementary information

**Additional file 1:** Additional figures and tables.

**Additional file 2:** Appendix protocol plant methods.

## Data Availability

The raw reads were deposited in Genbank with the Bioproject accession numbers PRJNA549137. Other datasets used for the study are available in the supplemental data or can be requested from the corresponding author on reasonable request.
