# Supplementary material for: A protocol for laser microdissection (LMD) followed by transcriptome analysis of plant reproductive tissue in phylogenetically distant angiosperms
Source: Plant Methods. 2019 Dec 16;15:151. doi: 10.1186/s13007-019-0536-3 (PMC6913016; doi:10.1186/s13007-019-0536-3)
Supplement: Supplementary file 1 — Additional file 1: Additional figures and tables. [file 13007_2019_536_MOESM1_ESM.pdf]

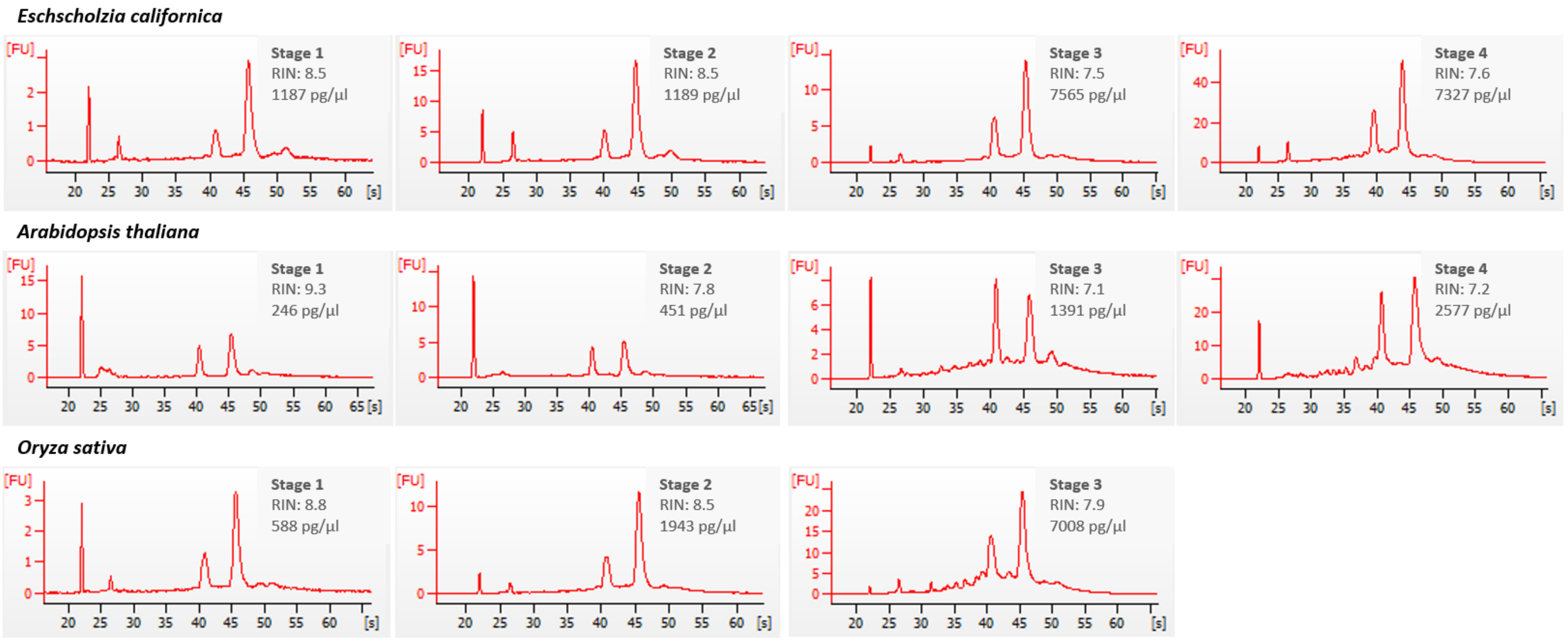

Supplemental Figure S1: Examples of electropherograms of one replicate of the RNA obtained by LMD of the three species and four (three for *O. sativa*) stages.

Table S1: Metadata of *A. thaliana* RNAseq showing sample ID with the corresponding stages [S1-S4]. Transcripts with a TPM < 5 are considered as genes present in the transcriptome.

**Sample_ ID**	**Species**	**Stage [Replica]**	**Total RNA [pg]**	**Total RNA [ng]**	**Quality [RIN]**	**Total area [µm]**	**Pg RNA / area (µm)**	**raw reads***	**trimmed reads***	**reads total [used for RNASeq]***	**reads mapped ***	**reads not mapped***	**Protein coding reads***	**Protein coding [%]**	**rRNA reads***	**rRNA reads [%]**	**intergenic reads***	**Transcripts TPM over 5**
**167-A1**	A. thaliana Col-0	S1-B	3654	3.654 [low]	8.7	84 [xs]	43,5	48,8 Mio	48,8 Mio	48,8 Mio	36,4 Mio	12,4 Mio	8,7 Mio	36.76	14,8 Mio	62.37	12,6 Mio	14809
**169-A1**	A. thaliana Col-0	S1-A	3444	3.444 [low]	9.3	91 [xs]	37,9	41,7 Mio	41,7 Mio	41,7 Mio	32,1 Mio	9,6 Mio	6,9 Mio	33.17	13,8 Mio	66.08	11,1 Mio	14861
**170-A1**	A. thaliana Col-0	S1-C	4648	4.648 [low]	8	98 [xs]	47,4	43,1 Mio	43,0 Mio	43,0 Mio	33,2 Mio	9,8 Mio	6,2 Mio	29.82	14,4 Mio	69.46	12,5 Mio	14908
**161-A1**	A. thaliana Col-0	S1-D	3164	3.164 [low]	9.1	84 [xs]	37,7	43,0 Mio	43,0 Mio	43,0 Mio	32,5 Mio	10,6 Mio	5,8 Mio	28.14	14,7 Mio	70.82	11,7 Mio	14441
**180-A2**	A. thaliana Col-0	S2-A	16968	16.968 [middle]	9.6	174 [s]	97,5	46,2 Mio	46,2 Mio	46,2 Mio	35,1 Mio	11,1 Mio	8,9 Mio	36.33	15,5 Mio	62.91	10,5 Mio	15049
**181-A2**	A. thaliana Col-0	S2-B	6314	6.314 [low]	7.8	132 [s]	47,8	45,9 Mio	45,9 Mio	45,9 Mio	34,3 Mio	11,6 Mio	6,1 Mio	27.53	15,8 Mio	71.7	12,3 Mio	14929
**182-A2**	A. thaliana Col-0	S2-C	9044	9.044 [low]	7.1	138 [s]	65,5	44,0 Mio	44,0 Mio	44,0 Mio	31,6 Mio	12,3 Mio	6,5 Mio	31.04	14,2 Mio	68.11	10,8 Mio	15425
**160-A2**	A. thaliana Col-0	S2-D	4746	4.746 low]	9.2	126 [s]	37,7	37,8 Mio	37,8 Mio	37,8 Mio	28,5 Mio	9,4 Mio	4,9 Mio	27.16	13,1 Mio	72.15	10,2 Mio	14757
**191-A3**	A. thaliana Col-0	S3-D	37114	37.114 [high]	7.2	528 [M]	70,3	42,9 Mio	42,9 Mio	42,9 Mio	32,1 Mio	10,8 Mio	4,9 Mio	23.1	16,3 Mio	76.38	10,8 Mio	14877
**192-A3**	A. thaliana Col-0	S3-A	19474	19.474 [middle]	7.1	384 [M]	50,7	58,7 Mio	58,7 Mio	58,7 Mio	44,7 Mio	13,9 Mio	10,0 Mio	31.42	21,6 Mio	68.02	12,9 Mio	14965
**193-A3**	A. thaliana Col-0	S3-B	22638	22.638 [middle]	7.2	440 [M]	51,4	52,4 Mio	52,4 Mio	52,4 Mio	40,3 Mio	12,1 Mio	8,1 Mio	28.78	19,9 Mio	70.65	12,1 Mio	14964
**194-A3**	A. thaliana Col-0	S3-C	32802	32.802 [high]	8.3	592 [M]	55,4	47,8 Mio	47,8 Mio	47,8 Mio	36,2 Mio	11,6 Mio	5,4 Mio	22.37	18,6 Mio	77.14	12,0 Mio	15037
**187-A4**	A. thaliana Col-0	S4-D	60676	60.676 [high]	8.3	2264 [XL]	26,8	50,6 Mio	50,6 Mio	50,6 Mio	38,0 Mio	12,5 Mio	5,1 Mio	18.88	21,6 Mio	80.68	11,2 Mio	14528
**188-A4**	A. thaliana Col-0	S4-A	36078	36.078 [high]	7.2	1756 [L]	20,5	57,0 Mio	57,0 Mio	57,0 Mio	43,1 Mio	13,9 Mio	5,6 Mio	18.44	24,4 Mio	81.15	13,0 Mio	15167
**189-A4**	A. thaliana Col-0	S4-B	42154	42.154 [high]	7.8	1872 [L]	22,5	54,7 Mio	54,7 Mio	54,7 Mio	42,1 Mio	12,5 Mio	6,4 Mio	22.08	22,6 Mio	77.47	12,9 Mio	15054
**190-A4**	A. thaliana Col-0	S4-C	64708	64.708 [very high]	8	2120 [XL]	30,5	52,0 Mio	52,0 Mio	52,0 Mio	39,6 Mio	12,4 Mio	6,0 Mio	21.69	21,6 Mio	77.9	12,0 Mio	14425
*** number referes to fragments**																		

Table S2: Metadata of *O. sativa* RNAseq showing sample ID with the corresponding stages [S1-S3]. Transcripts with a TPM $\leq$ 5 are considered as genes present in the transcriptome.

**Sample _ID**	**Species**	**Stage [Replica]**	**TOTAL RNA [pg]**	**TOTAL RNA [ng]**	**QUALITY [RIN]**	**TOTAL AREA [µm]**	**Pg RNA / area (µm)**	**raw reads***	**trimmed reads***	**reads total [used for RNASeq]***	**reads mapped ***	**reads not mapped ***	**protein_ coding reads***	**Protei n coding reads [%]**	**rRNA reads***	**rRNA reads [%]**	**interge nic reads***	**Transc ripts TPM over 5**
**R1-223**	*O. sativa spp. Dongjin*	S1-B	7056	7.056	8.80	112	63	40,2 Mio	40,2 Mio	40,2 Mio	28,4 Mio	11,8 Mio	6,9 Mio	35.66	12,4 Mio	63.76	9,0 Mio	15150
**R1-224**	*O. sativa spp. Dongjin*	S1-D	3624	3.624	8.50	96	37,8	30,0 Mio	30,0 Mio	30,0 Mio	21,6 Mio	8,4 Mio	4,8 Mio	35.66	9,8 Mio	66.73	6,9 Mio	15320
**R1-225**	*O. sativa spp. Dongjin*	S1-C	3756	3.756	8.50	96	39,1	35,3 Mio	35,3 Mio	35,3 Mio	24,3 Mio	10,9 Mio	4,1 Mio	35.66	12,1 Mio	74.25	8,1 Mio	15236
**R1-226**	*O. sativa spp. Dongjin*	S1-A	5496	5.496	8.40	104	52,9	39,3 Mio	39,3 Mio	39,3 Mio	28,2 Mio	11,0 Mio	6,9 Mio	35.66	12,5 Mio	63.93	8,6 Mio	15171
**R2-218**	*O. sativa spp. Dongjin*	S2-D	23316	23.316	8.50	168	138,8	41,3 Mio	41,3 Mio	41,3 Mio	31,6 Mio	9,7 Mio	5,7 Mio	35.66	15,7 Mio	72.98	10,1 Mio	15687
**R2-219**	*O. sativa spp. Dongjin*	S2-B	39672	39.672	8.70	240	165,3	38,7 Mio	38,7 Mio	38,7 Mio	27,9 Mio	10,8 Mio	5,4 Mio	35.66	13,8 Mio	71.54	8,6 Mio	15340
**R2-220**	*O. sativa spp. Dongjin*	S2-C	37728	37.728	8.90	204	184,9	41,0 Mio	41,0 Mio	41,0 Mio	29,9 Mio	11,1 Mio	5,4 Mio	35.66	15,1 Mio	73.47	9,3 Mio	15315
**R2-222**	*O. sativa spp. Dongjin*	S2-A	26640	26.64	8.90	168	158,6	37,0 Mio	37,0 Mio	37,0 Mio	27,0 Mio	9,9 Mio	4,5 Mio	35.66	13,7 Mio	75.13	8,8 Mio	15097
**R3-213**	*O. sativa spp. Dongjin*	S3-D	84096	84.096	7.90	1860	45,2	39,4 Mio	39,4 Mio	39,4 Mio	30,7 Mio	8,7 Mio	2,9 Mio	35.66	17,6 Mio	85.74	10,2 Mio	14372
**R3-214**	*O. sativa spp. Dongjin*	S3-C	104484	104.484	8.30	2106	49,6	32,8 Mio	32,8 Mio	32,7 Mio	25,5 Mio	7,3 Mio	2,6 Mio	35.66	14,6 Mio	84.66	8,2 Mio	14587
**R3-215**	*O. sativa spp. Dongjin*	S3-B	95580	95.58	8.20	2088	45,8	41,6 Mio	41,6 Mio	41,6 Mio	30,2 Mio	11,4 Mio	2,8 Mio	35.66	17,8 Mio	86.14	9,5 Mio	14660
**R3-230**	*O. sativa spp. Dongjin*	S3-A	121308	121.308	8.40	2328	52,1	41,9 Mio	41,9 Mio	41,9 Mio	31,6 Mio	10,3 Mio	4,5 Mio	35.66	17,6 Mio	79.33	9,5 Mio	14342
*** number refers to fragments**																		

Table S3: Metadata of *E. californica* RNAseq showing sample ID with the corresponding stages [S1-S4]. In addition, basic statistic from RNA-Seq is shown, including: raw reads, trimmed reads, total reads used for RNA-Seq, reads that mapped/ not mapped against the Reference Genome.

Sample _ID	Species	Stage [Replica]	TOTAL RNA [pg]	TOTAL RNA [ng]	QUALITY [RIN]	TOTAL AREA [µm]	Pg RNA / area (µm)	raw reads*	trimmed reads*	reads total [used for RNASeq]*	reads mapped*	reads not mapped*
**S1-24**	*E. californica*	S1-B	24458	24.458	7.80	84	291,17	46,5 Mio	46,5 Mio	46,5 Mio	29,6 Mio	16,9 Mio
**S1-18**	*E. californica*	S1-A	27160	27.16	7.50	112	242,50	33,0 Mio	33,0 Mio	33,0 Mio	20,5 Mio	12,4 Mio
**S1-19**	*E. californica*	S1-C	16618	16.618	8.50	77	215,82	36,5 Mio	36,5 Mio	36,5 Mio	23,5 Mio	12,9 Mio
**S2-13**	*E. californica*	S2-C	7854	7.854	7.40	335	23,44	37,6 Mio	37,6 Mio	37,6 Mio	19,2 Mio	18,4 Mio
**S2-22**	*E. californica*	S2-B	23254	23.254	7.00	381	61,03	31,6 Mio	31,6 Mio	31,6 Mio	16,7 Mio	14,9 Mio
**S2-23**	*E. californica*	S2-A	16646	16.646	8.50	410	40,60	38,8 Mio	38,8 Mio	38,8 Mio	25,4 Mio	13,5 Mio
**S3-9**	*E. californica*	S3-A	105910	105.91	7.50	1420	74,58	38,8 Mio	38,7 Mio	38,7 Mio	24,4 Mio	14,3 Mio
**S3-40**	*E. californica*	S3-C	6958	6.958	7.80	475	14,65	41,9 Mio	41,9 Mio	41,9 Mio	27,3 Mio	14,6 Mio
**S3-21**	*E. californica*	S3-B	18956	18.956	8.20	560	33,85	50,5 Mio	50,5 Mio	50,5 Mio	35,3 Mio	15,2 Mio
**S4-41**	*E. californica*	S4-B	12054	12.054	7.40	908	13,28	32,9 Mio	32,9 Mio	32,9 Mio	20,7 Mio	12,3 Mio
**S4-6**	*E. californica*	S4-A	102578	102.578	7.60	2364	43,39	36,3 Mio	36,2 Mio	36,2 Mio	23,1 Mio	13,2 Mio
**S4-26**	*E. californica*	S4-C	29358	29.358	7.20	1680	17,48	31,7 Mio	31,7 Mio	31,7 Mio	19,4 Mio	12,3 Mio
*** number refferes to fragments**												

Table S4: RNA quantity and quality measurements of the disqualified samples from two different species.

Sample_ID	Species	Stage	Concentration [pg/µl]	TOTAL RNA [pg]	TOTAL RNA [ng]	QUALITY [RIN]
**195 - A3**	*A. thaliana Col-0*	S3	4564,00	63896	63,90	7,00
**25 - P1**	*E. californica*	S1	2519,00	35266	35,27	6,30
**27 - P4**	*E. californica*	S4	960,00	13440	13,44	6,90
**29 - P3**	*E. californica*	S3	720,00	10080	10,08	6,90
**30 - P3**	*E. californica*	S3	928,00	12992	12,99	6,50
**13 - P1**	*E. californica*	S1	572,00	8008	8,01	6,10
**33 - P4**	*E. californica*	S4	3441,00	48174	48,17	6,90
**34 - P4**	*E. californica*	S4	2701,00	37814	37,81	6,60

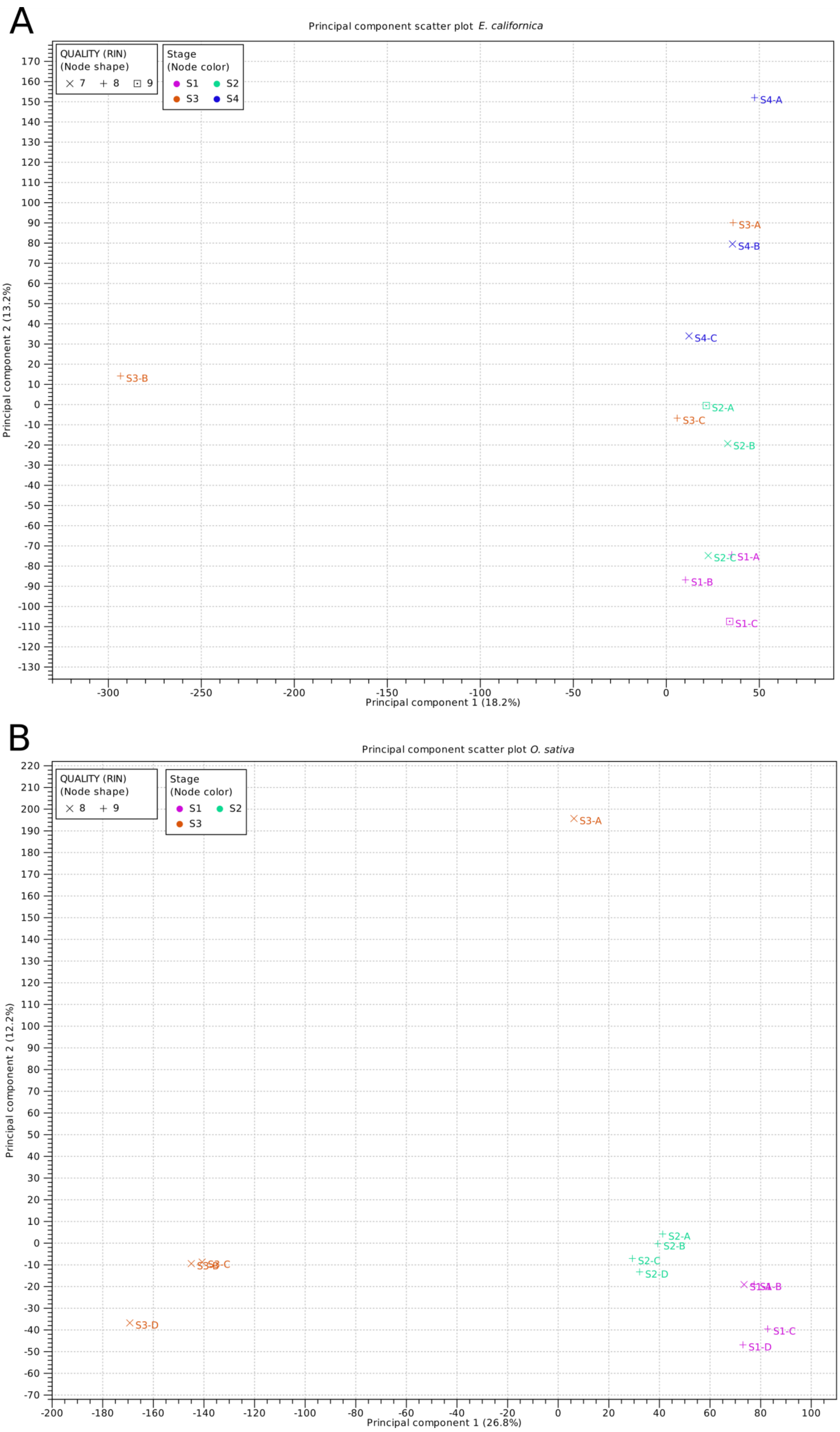

Figure S2: Principal component analyses of the *E. californica* [A] and *O. sativa* [B] RNA Seq samples. A, S1-S4 show the different developmental stages. A-C corresponds to the four replicas per stage, node shape corresponds to the rounded RIN values. B, S1-S3 show the different developmental stages. A-C corresponds to the four replicas per stage node shape corresponds to the rounded RIN values.

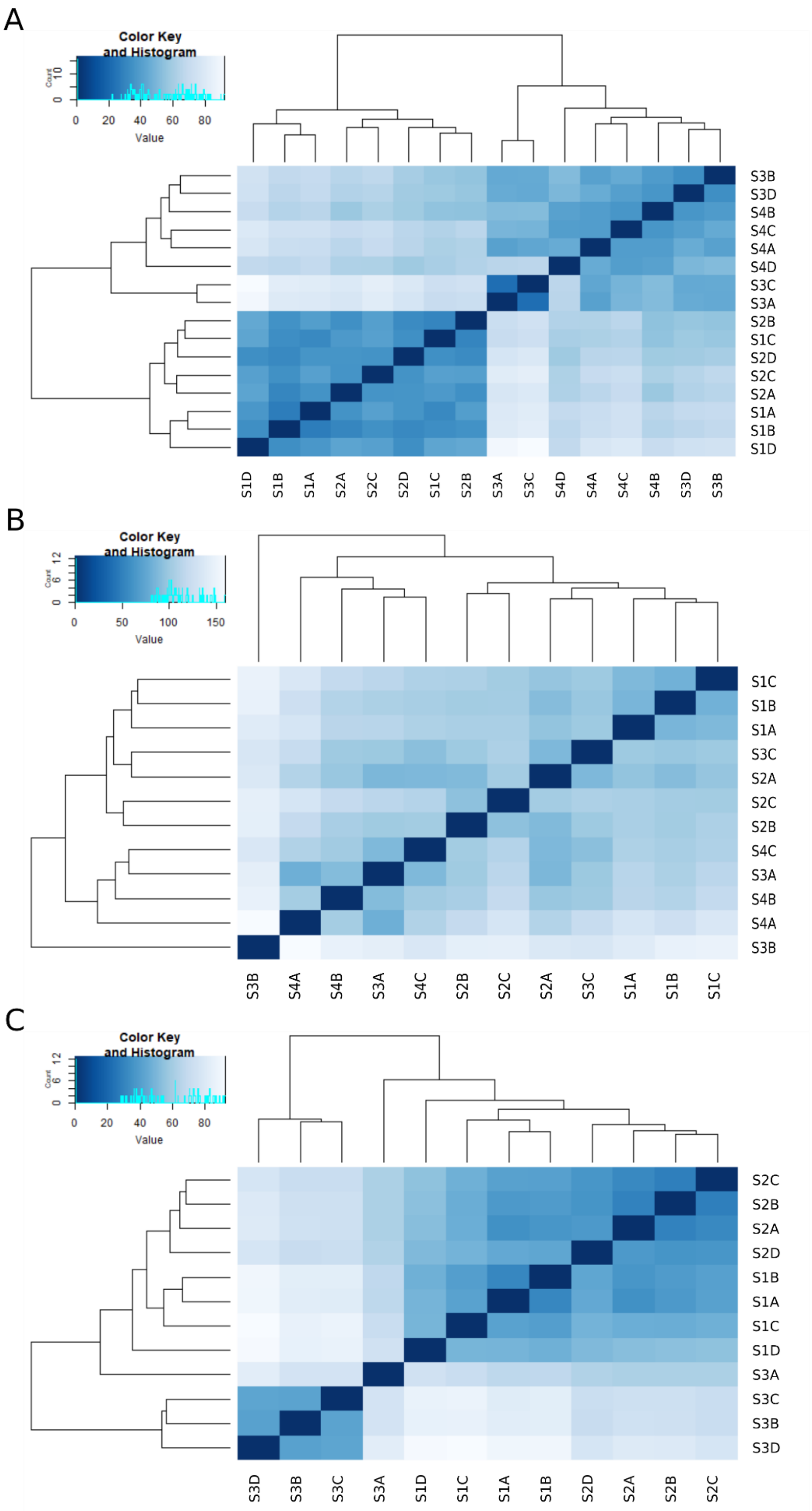

Figure S3: Sample distance correlation for replicas within each probe set was calculated. Dendogram at top and on side shows the relation of the replicas and samples. Samples were rlog transformed. A: *A. thaliana,* B: *E. californica,* C: *O. sativa.*

Table S5: Intron spanning primers for the four genes

Gene	F primer	R primer
**CRC**	CATCCTCGCGGTTGGGATAC	AAGGGTGAGGCTAACATGGC
**HAT1**	CCAAAACAGAAGAGCAAGGACA	AAAGTGTGGTCGGTGGACTC
**DDM1**	ACTGTTGGTCCCAAGTTCCC	TTCAACCTGTGGCCCTCATC
**CMT2**	CCACAGTCAGGCGTTATTGC	AGCACGAGAAACCGAGACTG

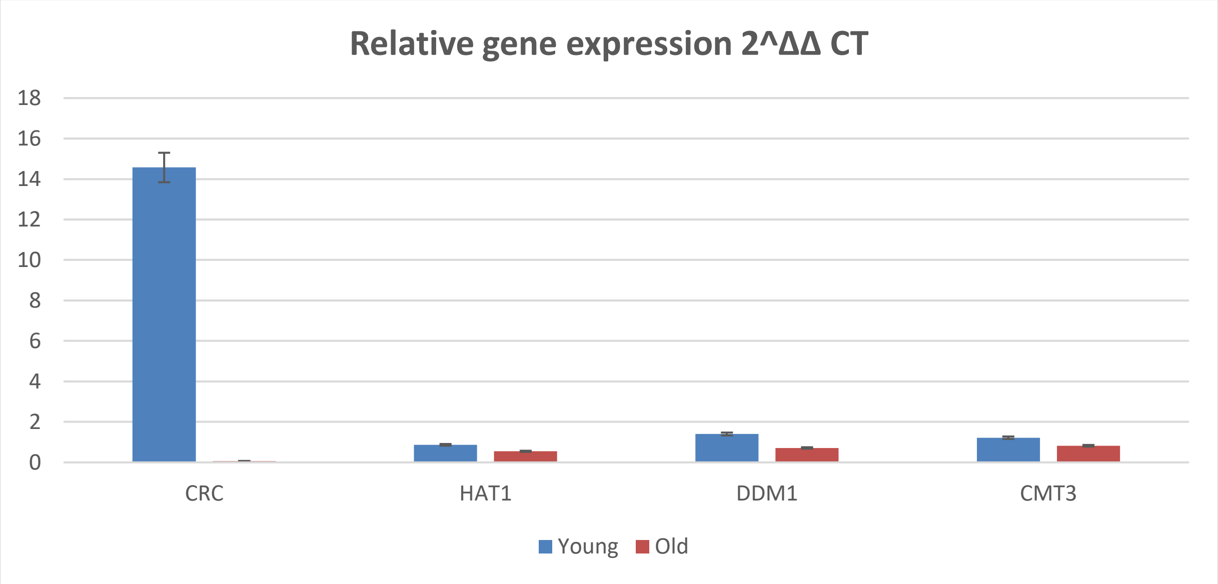

Figure S4: Expression analysis of carpel genes by qRT-PCR (Y-axis denotes relative units). Young: Stage 1, Old: Stage 4.

Table S6: *In silico* expression analysis based on TPM values

A. thaliana **transcriptomes**			**Klepikova atlas**		
	S1 carpel	S4 carpel		Young Flowers	Old Flowers
**CRC**	219,036826	1,05173537	**CRC**	65,46	0,23
**HAT1**	11,7137603	62,1998236	**HAT1**	22,55	44,38
**DDM1**	35,4033569	10,5592159	**DDM1**	18,92	9,07
**CMT3**	50,2581649	20,3983378	**CMT3**	27,69	17,58

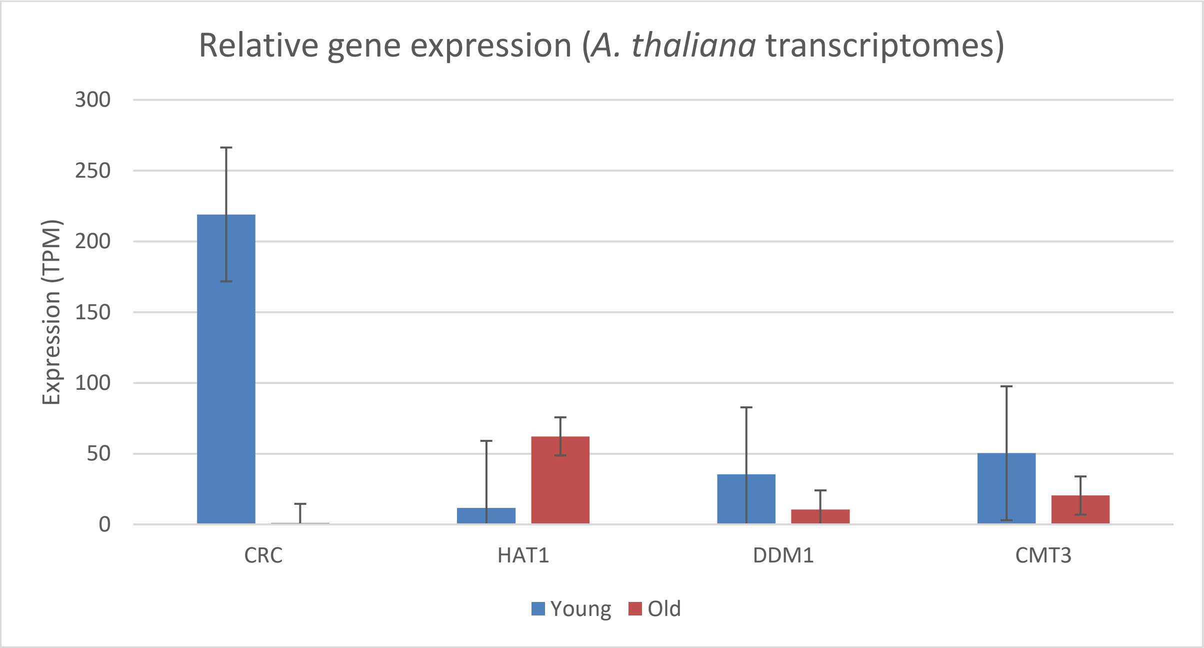

Figure S5: Relative expression of carpel genes as identified from this work's transcriptomes. Young: Stage 1, Old: Stage 4.

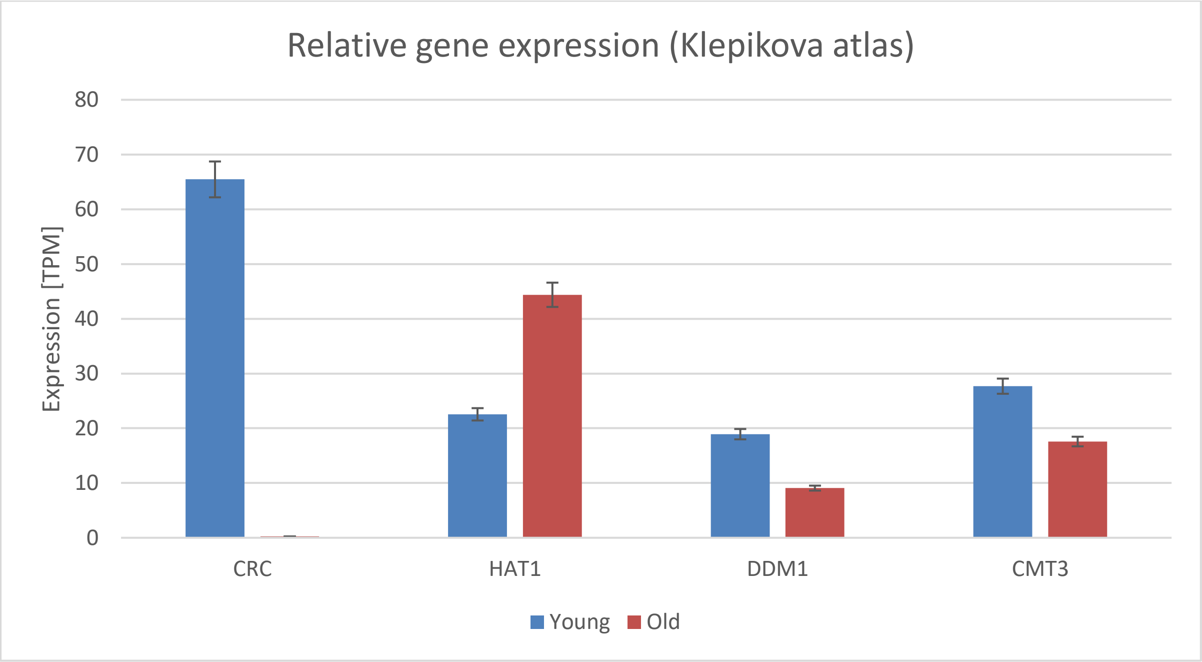

Figure S6: Relative expression of carpel genes as identified from Klepikova et al., 2016 [31]. Young "Carpels of the young flower." Old: "Carpels of the mature flower (before pollination)".

Table S7: Number of genes specific to single stages or patterns

Species	Name of pattern	Number of genes
A. thaliana	S1	143
	S1S2	629
	S1S2S3	514
	S1S2S4	97
	S1S3	34
	S1S3S4	56
	S1S4	6
	S2	225
	S2S3	56
	S2S3S4	195
	S2S4	31
	S3	427
	S3S4	444
	S4	74
O. sativa	S1	518
	S1S2	1147
	S1S3	181
	S2	328
	S2S3	382
	S3	789
E. californica	S1	507
	S1S2	299
	S1S2S3	547
	S1S2S4	407
	S1S3	289
	S1S3S4	484
	S1S4	225
	S2	688
	S2S3	326
	S2S3S4	963
	S2S4	289
	S3	895
	S3S4	711
	S4	837
	S1S2S3S4	13180
