# Supplementary material for: A protocol for laser microdissection (LMD) followed by transcriptome analysis of plant reproductive tissue in phylogenetically distant angiosperms
Source: Plant Methods. 2019 Dec 16;15:151. doi: 10.1186/s13007-019-0536-3 (PMC6913016; doi:10.1186/s13007-019-0536-3)
Supplement: Supplementary file 2 — Additional file 2: Appendix protocol plant methods. [file 13007_2019_536_MOESM2_ESM.pdf]

Appendix: Protocol

Reagents

- EAA (Ethanol (EtOH >99%), Acetic acid, 3:1)
- Sucrose
- 10X PBS buffer (80 g NaCl, 14,4 g $Na_2HPO_4 \cdot 7H_2O$, 2 g KCl, 2 g KH_2PO_4, bring to 1l with RNase free water, adjust the pH to 7.4)
- Tissue-Tek® O.C.T. Compound (Sakura Finetek Europe B.V., The Netherlands)
- Liquid nitrogen
- RNase AWAY™ Decontamination Reagent (Thermo Fisher Scientific, Waltham, Massachusetts, USA)
- PicoPure™ RNA Isolation Kit (Applied Biosystems™, Foster City, California, USA)
- RNase-free DNase (Qiagen, Hilden, Germany)
- Agilent bioanalyzer RNA 6000 Plant Pico Kit (Agilent Technologies, Santa Clara, California, USA)

Equipment

- Vacuum pump
- Freezers: -20 and -80 °C
- Undulating shaker
- Scalpels, blades and razor blades
- Fine-point forceps
- LMD film type 2 (Section-lab Co. Ltd., Hiroshima, Japan)
- Glass slides
- Frame slides, Steel frames, PET-membrane (Leica biosystems, Wetzlar, Germany)
- Falcon tubes (5, 20 ml)
- Embedding molds
- Cryostat (Leica CM1850, Leica biosystems, Wetzlar, Germany)
- Blades for cryomicrotome (Leica biosystems, Wetzlar, Germany)
- Leica DM6000 B microscope (Leica biosystems, Wetzlar, Germany), fitted with Crylas ftss 355-50 laser unit (CryLaS GmbH, Berlin, Germany)
- 0.2 ml collection tubes
- Agilent bioanalyzer (Agilent Technologies, Santa Clara, California, USA)
- Agilent RNA 6000 Pico Kit (Agilent Technologies, Santa Clara, California, USA)

- Stereo microscope
- Nugen Ovation® RNA-Seq System (PART NO. 7102) (NuGEN Technologies, San Carlos, California, United States)
- Illumina Nextera XT (Illumina Inc., San Diego, California, USA)

Protocol

The following protocol allows fixation, cryo-sectioning and laser microdissection followed by RNA extraction and RNAseq of floral tissue from diverse species (*Arabidopsis thaliana*, Brassicaceae; *Eschscholzia californica*, Papaveraceae; *Oryza sativa*, Poaceae). We expect this protocol to be useful for a much broader range of species. The procedure was optimised to ensure high structural integrity of developing flowers for cryo-sectioning and laser-microdissection. The cryoprotective steps of the protocol are based on a modified protocol by Martin et al. (2016). The steps of cryo-sectioning are based on a modified protocol by Kawamoto & Kawamoto (2014). Generally, to ensure an optimal yield of intact RNA, we recommend to work in aseptic conditions when possible and treat all working surfaces and tools with RNase AWAY.

1. Fixation and cryoprotection: For small, heterogenous samples fixation and cryoprotection are recommended to achieve adequate histological quality for the following steps of the protocol. For some homogenous samples these steps can be omitted to speed up the process (then start with the embedding step.)

A. Measure, document and harvest fresh flower buds with a clean scalpel and immediately submerge them in a falcon tube filled with ice cold EAA. Subsequent steps have to be carried out on ice. For large samples we recommend cutting or removing the outer layers to enhance the penetration of the fixative and cryoprotective solutions.

A. Apply vacuum (300-400 mbar) for 10 minutes to enhance penetration of the EAA solution.

B. Discard EAA and move the samples to a new falcon tube filled with 10% (w/v) sucrose solution prepared with PBS buffer.

C. Apply vacuum for 20 minutes. Samples will first float in the solution but will slowly start sinking as the solution is penetrating the tissues. Place the falcon tube with the samples on an undulating shaker in a cold room or a refrigerator and let the samples incubate for at least one hour with a low setting (larger samples might take a longer time). Make sure that the samples are moving freely inside the tube.

D. Replace the solution with 20% sucrose solution prepared with PBS buffer and repeat the vacuuming step of 20 minutes. Repeat the incubation step with the undulating shaker for one hour. Once the buds have sunk, they are ready to for embedding.

2. Embedding: Trim as much excess material as possible around the target tissue. This prevents subsequent dislocation of structures during the cryo-sectioning.

A. Move one to three samples similar in size to embedding moulds and try to remove as much sucrose solution as possible. Pour the O.C.T. compound on top of the buds to submerge them completely. Move the buds around the mould with forceps to ensure that the layer of sucrose solution is completely replaced with O.C.T. compound. Observe the buds under a stereoscope and move them into horizontal orientation in the bottom of the mould.

B. Freeze at -20 °C for 15-30 minutes. Once the samples are frozen, they can be placed in -80 °C for long term storage.

3. Sectioning: Before sectioning, adjust the cryochamber to -20 °C and the sample holder to -25 °C. Move the required instruments to the cryochamber and let them acclimate for 10 minutes (warm instruments and surfaces can easily thaw the O.C.T. compound).

A. Apply a piece of filter paper to the back of the frozen sample block with a drop of fresh O.C.T. medium and let it freeze in the cryostat. Applying filter paper helps in removal of the sample after sectioning. To adhere the sample to the sample base, apply a few drops of water on the base and quickly attach the sample to the base by the filter paper. The water will quickly solidify and attach the block to the sample base. Attach the sample base with the frozen sample block to the pre-frozen sample holder and tighten it firmly. Make sure the block is in parallel to the sample holder to allow accurate sectioning (Fig. 1L).

B. Trim and discard the excess O.C.T. compound and outer, unwanted layers by cutting the sample block with 20 µm section thickness. When closing in to the desired depth, take a sample section with 10 µm layer thickness, adhere them to a warm glass slide and observe their histological quality under a microscope.

C. When the desired depth and quality has been reached, take a cut pre-frozen piece of LMD film type 2, peel off the protective paper and adhere it to the section. Lightly press the LMD film with a gloved finger for 5-10 seconds to ensure proper adherence. Cut through the section with a slow, constant speed. While cutting press the section down with the help of a fine brush to prevent folding or wrinkling of the sample. (Fig. 1M)

D. Cut 4-12 sections depending on the size of the samples and adhere them to empty frame slides.

E. (optional) To remove excess OCT medium and moisture in the sample sections, let the samples thaw in room temperature for 30 seconds. After thawing completely submerge the films to a container containing 70% ethanol. Applying vacuum will further facilitate penetration of the ethanol.

4. Laser-microdissection: The dissecting microscope requires careful calibration to optimize target fragment dissection and collection.

Set laser control settings to:

- Power: 85
- Speed: 5
- Specimen balance: 10

The film needs to be fully dried before starting the laser-assisted cutting, because moisture in the films may lead to warping of the membrane during cutting or fragments adhering to the film. Samples can be observed and documented while the films are drying (some features in the sections can be more easily perceived before drying).

A. Attach two sample collection tubes to the sample collection tray: one for the samples and another for discarding unwanted material.

B. Locate the samples and make sure the structures of interest are intact.

C. Mark the elements of the unwanted tissue and cut them out into the collection tube for discarded material.

D. Mark the elements of the desired tissue. Before starting the cut, fill the collection tube with 20 µl of extraction buffer (PicoPure™ RNA Isolation Kit). Cut out the samples and make sure that the target tissue has fallen into the collection tube. Dissecting large samples in several smaller pieces may help to avoid the cut pieces from adhering to the surrounding film. Carefully detach the collection tube from the tray and close the tube. Vortex the tube for one minute and submerge the tubes in liquid nitrogen for further break down of protective cell structures.

5. RNA extraction and quality/quantity measurements

A. Continue with RNA extraction using the PicoPure™ RNA Isolation Kit according to the manufacturer's instructions or store the samples at -80 °C. Multiple samples can be pooled together for a single extraction to increase the RNA concentration of the eluate. RNA from samples with low amount of extracted tissue can additionally eluted with low amounts of buffer (12 µl) and in several consecutive elution steps. We recommend to use at least a combined area of 100 μm^2 for a section thickness of 10 µm to ensure RNA extraction of sufficient quantity (the success of the extraction might vary between target tissues and species).

B. Measure the quantity and quality of the samples using the Agilent bioanalyzer with Agilent RNA 6000 Pico Kit. Samples below 500 pg/µl should be measured twice to ensure the accuracy of the measurements.

6. Library preparation and sequencing

RNA samples were pre-amplified using Nugen Ovation® RNA-Seq System suitable for an input of 500 pg - 100 ng of RNA and the cDNA generated was used for library preparation. Amplification and cDNA generation was conducted according to the manufacturer's instructions.

Library preparation was conducted with the Illumina Nextera XT kit suitable for 1ng DNA. The cDNA was measured before the preparation with Qubit and normalized to 0,2ng/µl. 5µl of dilution was used for the library preparation. Libraries were prepared according to the manufacturer's instructions. Generated libraries were sequenced with Sequencing HiSeq 4000. 9 samples were sequenced per lane generating approximately 33Mio reads per sample. Paired-end sequencing was used for each sample. Read length was approximately 76 bp.

7. Assembly/RNA-seq/Heatmap and PCA

Raw reads of paired end sequencing data was important to CLC genomics workbench version 11.0.1. CLC Genomics workbench was used for quality test, trimming, RNA-seq and PCA analysis using the default parameters (Supplemental table 1-4). Additionally, a metadata table, including several parameters (RNA Quality, number of raw reads etc) was created to explain the PCA.
